# Enlicitide, a Novel Oral Macrocyclic Peptide PCSK9 Inhibitor for Lipid Lowering: A Systematic Review and Meta-Analysis

**DOI:** 10.3390/jcdd13080398

**Published:** 2026-08-20

**Authors:** Burcu Yagmur, Elif Ijlal Cekirdekci

**Affiliations:** Department of Cardiology, University of Kyrenia, Kyrenia 99320, Cyprus; elifijlal.cekirdekci@kyrenia.edu.tr

**Keywords:** enlicitide, MK-0616, PCSK9 inhibitor, oral lipid-lowering therapy, LDL-C, familial hypercholesterolemia, meta-analysis

## Abstract

Enlicitide (MK-0616) is an oral proprotein convertase subtilisin/kexin type 9 (PCSK9) inhibitor for the treatment of hypercholesterolemia. We conducted the first systematic review and meta-analysis evaluating the efficacy and safety of enlicitide across randomized controlled trials enrolling adults with hypercholesterolemia or heterozygous familial hypercholesterolemia (HeFH). The primary outcome was the baseline-to-endpoint percentage change in low-density lipoprotein cholesterol (LDL-C); secondary outcomes included apolipoprotein B (ApoB), non-high-density lipoprotein cholesterol (non-HDL-C), lipoprotein(a) [Lp(a)], and safety. Four randomized controlled trials involving 3894 participants were included. Enlicitide achieved a substantial reduction in LDL-C (mean difference [MD]: −55.78 percentage points; 95% confidence interval [CI]: −61.15 to −50.42; *p* < 0.0001; I^2^ = 99.58%), with sustained efficacy demonstrated in the newly available 52-week Phase 3 CORALreef data. Significant reductions were also observed in ApoB (MD: −47.95 pp; 95% CI: −51.22 to −44.67), non-HDL-C (MD: −49.42 pp; 95% CI: −54.23 to −44.60), and Lp(a) (MD: −22.70 pp; 95% CI: −28.62 to −16.78). The pooled incidence of any adverse event was not statistically significant (event rate: 0.21; *p* = 0.087), and treatment discontinuation due to adverse events was uncommon (event rate: 0.007). Enlicitide demonstrated substantial lipid-lowering efficacy with an acceptable safety profile. The ongoing CORALreef Outcomes trial will determine whether these lipid improvements translate into reductions in major adverse cardiovascular events.

## 1. Introduction

Elevated levels of low-density lipoprotein cholesterol (LDL-C) stand as the most extensively documented, modifiable driver of atherosclerotic cardiovascular disease (ASCVD), which continues to reign as the leading cause of mortality worldwide [1]. Despite the widespread availability of statins and adjunctive oral agents such as ezetimibe and bempedoic acid, a substantial proportion of high-risk patients fail to achieve guideline-recommended LDL-C targets—owing to statin intolerance, insufficient efficacy of oral non-statin therapies, or suboptimal adherence [2,3].

Injectable proprotein convertase subtilisin/kexin type 9 (PCSK9) monoclonal antibodies, including evolocumab and alirocumab, are primarily used in patients with familial hypercholesterolemia (FH) and those at very high risk of ASCVD [4]. These agents reduce LDL-C by approximately 55–60% on top of background statin therapy, with landmark clinical trials confirming a corresponding 15% reduction in the risk of major adverse cardiovascular events [4,5,6]. However, their real-world uptake remains limited by the need for subcutaneous self-injection; cold-chain storage; and, in many healthcare systems, restrictive reimbursement policies.

Enlicitide (MK-0616) is a novel oral macrocyclic peptide inhibitor of PCSK9 currently in Phase 3 development [7]. Unlike monoclonal antibodies, it is administered orally and directly binds PCSK9, preventing LDL receptor degradation. Data from the Phase 2b dose-ranging study and the Phase 3 CORALreef program have demonstrated significant LDL-C lowering across diverse populations, including heterozygous familial hypercholesterolemia (HeFH) and non-familial hypercholesterolemia [8,9]. To date, however, no systematic review or meta-analysis has quantitatively synthesized the available RCT evidence, including the most recent 52-week Phase 3 data.

Enlicitide received US Food and Drug Administration approval on 16 July 2026, becoming the first oral PCSK9 inhibitor approved for lowering LDL-C in adults with primary hypercholesterolemia (including heterozygous familial hypercholesterolemia) as an adjunct to diet and maximally tolerated statin therapy. This regulatory milestone increases the timeliness and clinical relevance of a quantitative synthesis of its efficacy and safety.

We therefore conducted this systematic review and meta-analysis to provide the first comprehensive pooled estimate of the efficacy and safety of enlicitide—incorporating 52-week follow-up data from the two pivotal Phase 3 CORALreef trials—to inform clinical practice, guideline development, and future research priorities.

## 2. Materials and Methods

### 2.1. Study Design and Registration

This systematic review and meta-analysis was conducted and reported in accordance with the Preferred Reporting Items for Systematic Reviews and Meta-Analyses (PRISMA 2020) statement [10]. The protocol was prospectively registered in the International Prospective Register of Systematic Reviews (PROSPERO; registration number CRD420261437716) [11].

### 2.2. Eligibility Criteria

Eligibility was defined using the PICOS framework. Population: Adults (≥18 years) with hypercholesterolemia or HeFH. Intervention: Enlicitide (MK-0616) at any dose and frequency. Comparator: Not applicable to the pooled effect measure. Each trial’s comparator arm (placebo, ezetimibe, bempedoic acid, or their combination) is reported only as a descriptive trial characteristic and was not included in the pooled analyses. Outcomes: Within-arm percent change in LDL-C from baseline (primary), with secondary outcomes comprising percent changes in apolipoprotein B (ApoB), non-high-density lipoprotein cholesterol (non-HDL-C), lipoprotein(a) [Lp(a)], total cholesterol, triglycerides, and HDL-C, and safety event rates. Study design: Randomized controlled trials with a follow-up duration of ≥4 weeks.

Studies were excluded if they enrolled exclusively pediatric populations (<18 years), healthy volunteers in pharmacokinetic/pharmacodynamic-only trials, patients with homozygous FH, or those with severe hypertriglyceridemia (triglycerides ≥ 400 mg/dL). Single-arm trials, uncontrolled extension studies, trials with a follow-up duration <4 weeks, and studies that lacked an extractable LDL-C efficacy outcome were also excluded, alongside non-randomized designs, case reports, and conference abstracts lacking sufficient methodological detail.

### 2.3. Information Sources and Search Strategy

A systematic literature search was performed across four electronic databases—PubMed, Scopus, Web of Science, and the Cochrane Library—on 29 June 2026, with no restriction on publication date or language. The following core search concept was applied and adapted to each database’s syntax:

(“enlicitide” OR “MK-0616” OR “MK0616” OR “enlicitide decanoate” OR “oral PCSK9 inhibitor” OR “macrocyclic peptide PCSK9”) AND (“randomized controlled trial” OR “randomised” OR “RCT” OR “clinical trial” OR “double-blind” OR “placebo-controlled” OR “placebo”) AND (“LDL” OR “low-density lipoprotein” OR “cholesterol” OR “hypercholesterolemia” OR “hyperlipidaemia” OR “familial hypercholesterolemia”).

Gray literature and additional records were sought through the reference lists of included studies and relevant reviews, and through the scientific session archives of the American Heart Association and the European Society of Cardiology (2022–2026). All identified records were imported into Covidence systematic review software (available at: https://www.covidence.org/ (accessed on 8 July 2026)) for de-duplication, screening, and full-text assessment [12].

### 2.4. Study Screening and Selection Process 

Following automated and manual de-duplication in Covidence, title and abstract screening and subsequent full-text eligibility assessments were performed by the primary reviewer (B.Y.) and independently verified by a second reviewer (E.I.C.). Disagreements at each stage were resolved through discussion and consensus. The complete selection process, including the numbers of records identified, screened, sought for retrieval, assessed for eligibility, and excluded (with reasons), is presented in the PRISMA 2020 flow diagram [10]. (The PRISMA 2020 Main Checklist is in the Supplementary Materials).

### 2.5. Data Extraction

Data were extracted by the primary reviewer (B.Y.) using a pre-specified, standardized extraction form and verified by the second reviewer (E.I.C.). Extracted variables comprised trial characteristics (author, year, trial name, phase, design, country, funding source, follow-up duration), participant demographics (sample size, baseline lipids, HeFH status, background therapy), intervention doses, and quantitative primary/secondary outcome data. For CORALreef Lipids (NCT05952856) [13] and CORALreef HeFH (NCT05952869) [9], 52-week follow-up data were used in preference to shorter-term assessments, as these represented the longest available and most clinically relevant time point. For the remaining studies, the primary-endpoint assessment time point reported in the original publication was used.

### 2.6. Risk of Bias Assessment

Methodological quality was evaluated independently by two reviewers using the Cochrane Risk of Bias 2 (RoB 2) tool [14]. This framework assesses five core domains: the randomization process, deviations from intended interventions, missing outcome data, measurement of the outcome, and selection of the reported result. Discrepancies were resolved via consensus. For each trial, individual domains were classified as posing low risk, raising some concerns, or carrying high risk of bias, which subsequently informed the final, integrated risk-of-bias judgment. Because all included trials were sponsored by a single manufacturer (Merck & Co., Inc., Rahway, NJ, USA), this single-sponsor evidence base was pre-specified as a potential source of bias and was explicitly evaluated qualitatively under the publication bias domain. Small-study effects and formal publication bias testing via funnel plots were pre-specified only if ten or more trials were included; otherwise, qualitative assessment was planned.

### 2.7. Certainty of Evidence (GRADE)

The certainty of evidence for each primary and secondary outcome was graded independently using the Grading of Recommendations, Assessment, Development, and Evaluations (GRADE) guidelines [15]. Evidence profiles were generated using the GRADEpro Guideline Development Tool (GRADEpro GDT; Evidence Prime Inc., Hamilton, ON, Canada) [16]. Criteria for downgrading included risk of bias, inconsistency, indirectness, imprecision, and publication bias. Industry sponsorship was systematically evaluated under the publication bias domain to avoid double-counting limitations already captured in the RoB 2 assessment.

### 2.8. Statistical Analysis

All meta-analyses were performed using Comprehensive Meta-Analysis software (version 4; Biostat, Englewood, NJ, USA) [17] and Review Manager (RevMan, version 5.4; The Cochrane Collaboration, Copenhagen, Denmark) [18]. For continuous lipid outcomes, the effect measure for each trial was the within-arm (single-group) percentage change in the lipid parameter from baseline to the designated time point in the enlicitide-treated arm; comparator arms (placebo, ezetimibe, bempedoic acid, or their combination) were not included in the pooled analyses and are reported only as descriptive trial characteristics. Because only enlicitide-arm change-from-baseline values were pooled, all included estimates represent the same within-treatment quantity irrespective of each trial’s comparator. The pooled effect was expressed as the weighted mean of these within-arm percentage changes with 95% confidence intervals (CIs). Safety outcomes were pooled as event rates with 95% CIs. A random-effects model using the DerSimonian and Laird method was applied throughout to account for anticipated clinical and methodological heterogeneity in populations, doses, comparators, and follow-up durations [19].

Statistical heterogeneity was quantified using the Cochran Q statistic (significance threshold *p* < 0.10) and the I^2^ statistic, interpreted as low (<25%), moderate (25–50%), substantial (50–75%), or considerable (>75%). Sensitivity was evaluated using leave-one-out analyses. Pre-specified subgroup analyses were conducted according to diagnosis (HeFH vs. non-familial hypercholesterolemia), enlicitide dose, and concomitant statin use, where sufficient studies were available for analysis. As an exploratory analysis, a random-effects (method-of-moments) meta-regression of percent LDL-C change on enlicitide dose was performed to evaluate dose-dependency and the extent to which dose accounted for between-study heterogeneity. Because the lower and higher dose levels (6, 12, 18, and 30 mg) were contributed by the separate randomized dose arms of a single Phase 2b trial, whereas the 20 mg dose derived exclusively from the Phase 3 trials, the dose and study phase were substantially confounded. Consequently, this meta-regression was interpreted strictly as hypothesis-generating rather than confirmatory. A two-tailed *p*-value <0.05 was considered statistically significant unless otherwise specified.

## 3. Results

### 3.1. Study Selection

**Figure 1:**
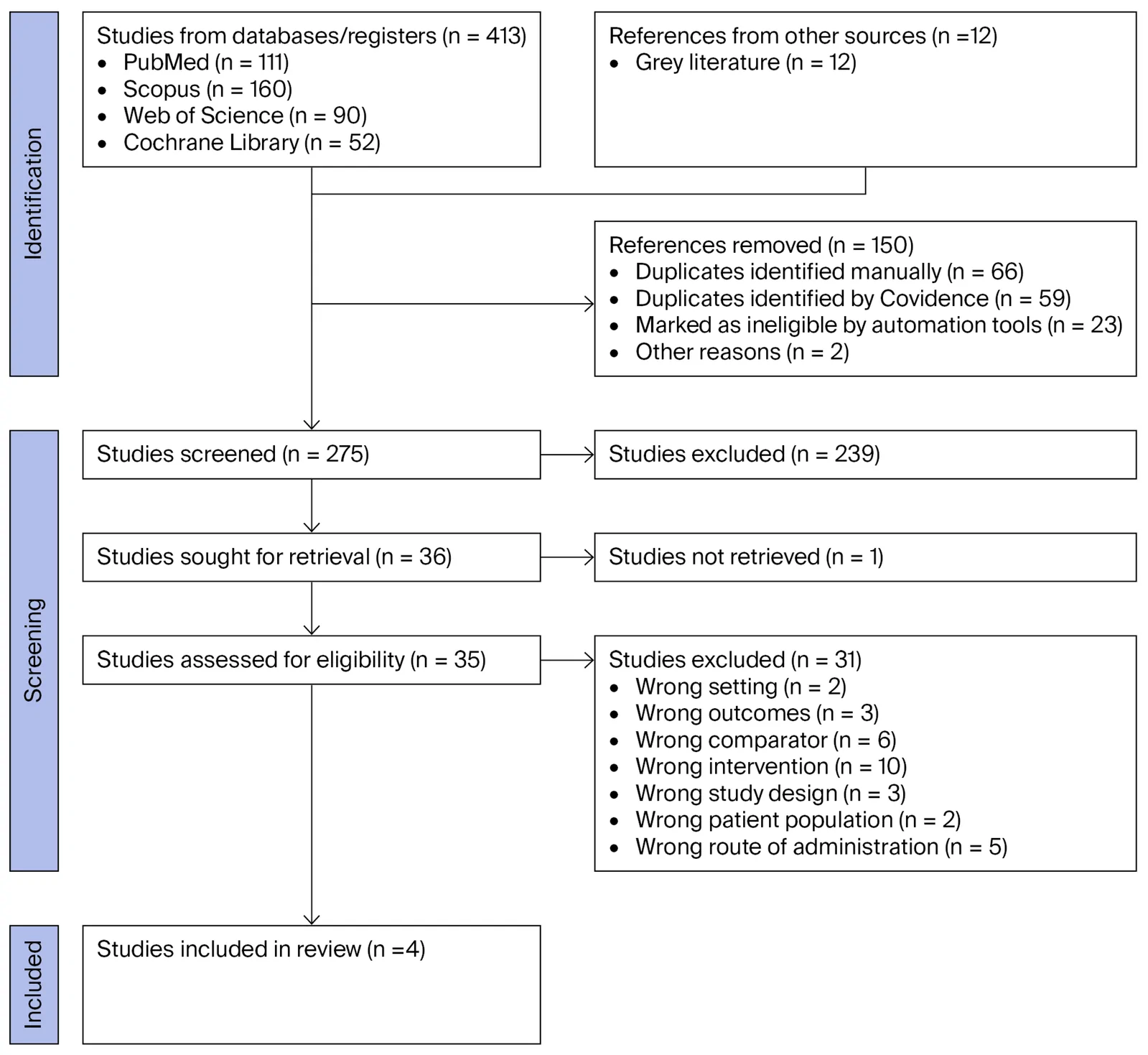
Preferred Reporting Items for Systematic Reviews and Meta-Analysis (PRISMA) flow diagram for the study selection process.

The systematic search yielded 413 database records and 12 gray literature records. Following the removal of 150 duplicates, 275 records underwent title and abstract screening. Of these, 239 records were excluded. Full-text evaluation of the remaining 35 studies resulted in the exclusion of 31 papers for specific reasons (wrong intervention, *n* = 10; wrong comparator, *n* = 6; wrong route of administration, *n* = 5; wrong outcomes, *n* = 3; wrong study design, *n* = 3; wrong setting, *n* = 2; wrong patient population, *n* = 2). Ultimately, four RCTs meeting all selection criteria were included in this meta-analysis (Figure 1).

A graphical summary integrating the evidence base (four RCTs; 3894 participants), the mechanism of oral PCSK9 inhibition, the pooled within-arm reductions in LDL-C, ApoB, non-HDL-C, and Lp(a), and the principal limitations—including that cardiovascular outcome benefit remains under investigation in the ongoing CORALreef Outcomes trial—is provided in Figure 1.

### 3.2. Characteristics of Included Studies

The four included trials enrolled a total of 3894 randomized participants and comprised one Phase 2b dose-ranging study [8] and three Phase 3 trials from the CORALreef program: CORALreef Lipids [13], CORALreef HeFH [9], and CORALreef AddOn [20]. All trials were sponsored by Merck & Co., Inc. (Rahway, NJ, USA). Three trials used a placebo comparator, while CORALreef AddOn employed an active-comparator design against bempedoic acid, ezetimibe, and their combination. Baseline LDL-C ranged from 92.5 mg/dL in CORALreef AddOn to 119 mg/dL in the HeFH population. Background statin use was near-universal in the Phase 3 trials (96.6–100%). Enlicitide was administered orally once daily at 20 mg in all Phase 3 trials, and at 6–30 mg in the Phase 2b dose-ranging study. The trial characteristics are summarized in Table 1.

### 3.3. Primary Outcome: LDL-C Percent Change from Baseline

**Figure 2:**
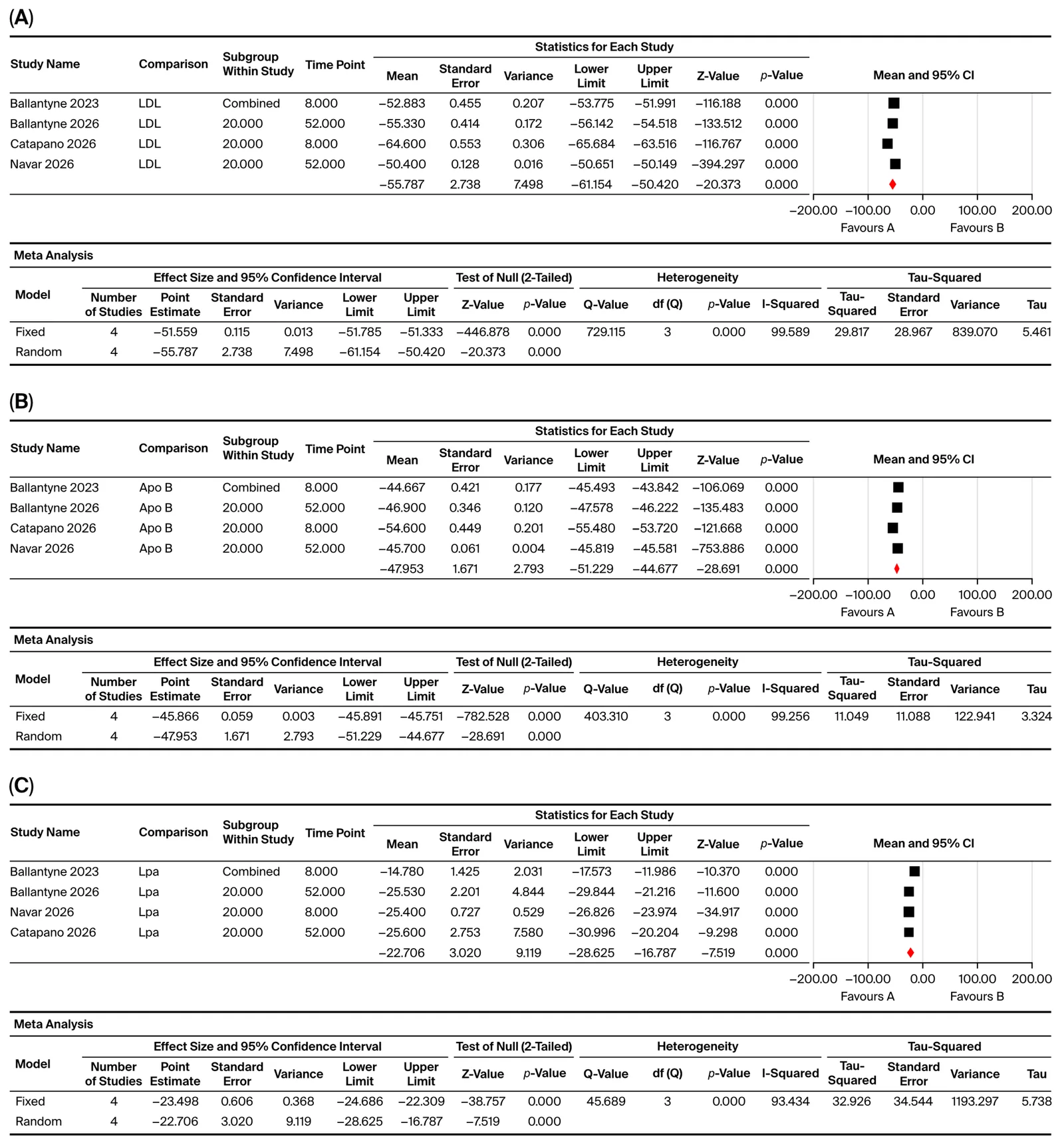

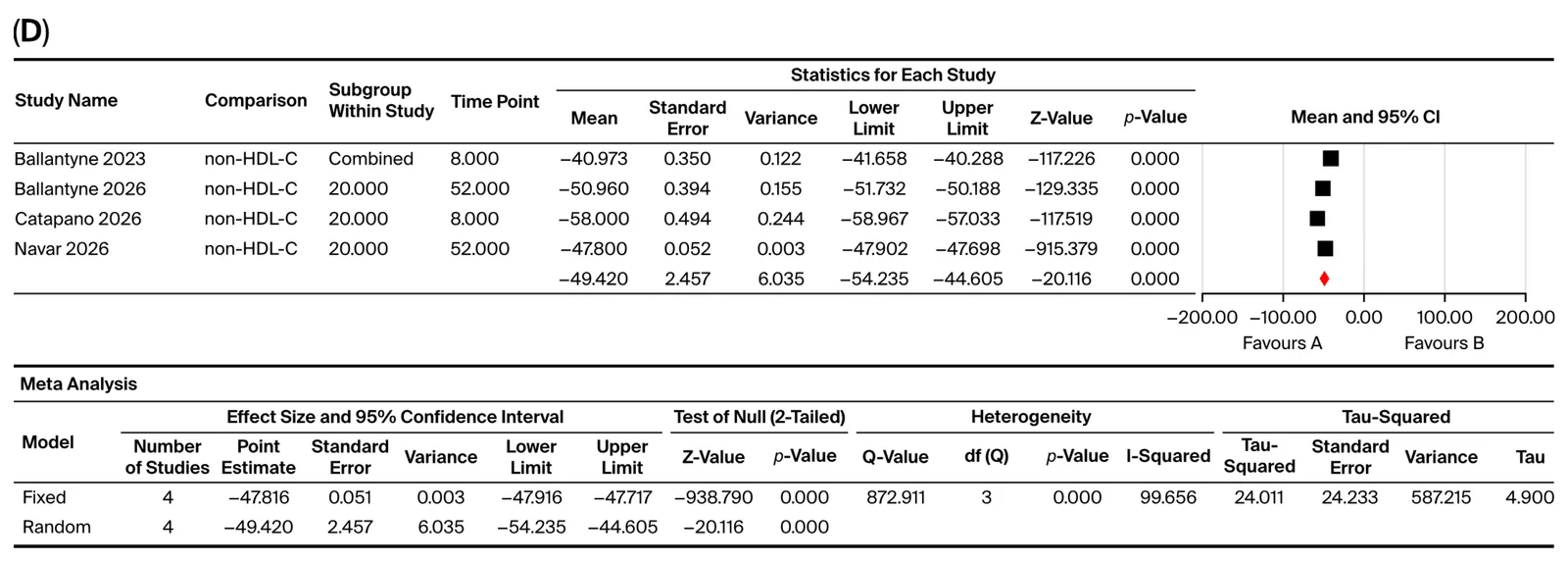
Forest plots of pooled percent change from baseline in lipid parameters with enlicitide [8,9,13,20]. (**A**) Low-density lipoprotein cholesterol (LDL-C); (**B**) apolipoprotein B (ApoB); (**C**) lipoprotein(a) [Lp(a)]; (**D**) non-high-density lipoprotein cholesterol (non-HDL-C). For each panel, the upper section displays individual study estimates with corresponding enlicitide dose (mg), assessment time point (weeks), mean difference, standard error, variance, 95% confidence interval, Z-value, and *p*-value; black squares represent individual study effect estimates, with square size proportional to study weight, and horizontal lines denote 95% confidence intervals. The red diamond represents the pooled random effects estimate. The lower section of each panel summarizes the overall effect size, test of the null hypothesis, heterogeneity statistics (Cochran Q, degrees of freedom, *p*-value, I^2^), and between-study variance (τ^2^, standard error, variance, τ).

Pooled analysis demonstrated that enlicitide produced a statistically significant and clinically meaningful reduction in LDL-C from baseline within the enlicitide arm (MD: −55.78 percentage points; 95% CI: −61.15 to −50.42; *p* < 0.0001). Considerable heterogeneity was observed (I^2^ = 99.58%, *p* < 0.0001), consistent with differences in enlicitide dose, patient populations, and background therapies. Leave-one-out sensitivity analysis confirmed the directional stability of this estimate (with I^2^ ranging from 65% to 90% following sequential omission of individual studies), indicating that no single trial disproportionately influenced the pooled outcome (Figure 2).

Efficacy data from the two largest Phase 3 trials—CORALreef Lipids and CORALreef HeFH—were derived from 52-week follow-up assessments, representing the longest available duration of enlicitide treatment in a randomized controlled setting. The sustained magnitude of LDL-C reduction at 52 weeks indicates that the lipid-lowering effect of enlicitide is durable and does not attenuate with prolonged treatment—a finding of considerable importance for long-term cardiovascular risk management.

### 3.4. Secondary Efficacy Outcomes

#### 3.4.1. Apolipoprotein B

Enlicitide significantly reduced ApoB levels with considerable heterogeneity (MD: −47.95 percentage points; 95% CI: −51.22 to −44.67; *p* < 0.0001; I^2^ = 99.25%, *p* < 0.0001). Leave-one-out analysis confirmed robustness, although heterogeneity remained substantial (I^2^ range, 78–94%) (Figure 2).

#### 3.4.2. Non-HDL Cholesterol

A significant reduction in non-HDL-C was observed (MD: −49.42 percentage points; 95% CI: −54.23 to −44.60; *p* < 0.0001; I^2^ = 99.65%; *p* < 0.0001), consistent in magnitude with the primary LDL-C finding (Figure 2).

#### 3.4.3. Lipoprotein(a)

Enlicitide significantly reduced Lp(a) (MD: −22.70 percentage points; 95% CI: −28.62 to −16.78; *p* < 0.0001; I^2^ = 93.43%; *p* < 0.0001). Although heterogeneity remained considerable, the consistent and substantial reduction in Lp(a)—a lipoprotein largely unresponsive to statins—is of clinical interest (Figure 2).

### 3.5. Safety Outcomes

The pooled safety analysis demonstrated a favorable tolerability profile for enlicitide. The event rate for any serious adverse event was 0.042 (95% CI: 0.017–0.101; *p* < 0.0001), reflecting a low absolute burden of serious harm. For any adverse event, the pooled event rate was 0.21 (95% CI: 0.05–0.54; *p* = 0.087), which did not reach statistical significance, indicating a low pooled incidence of adverse events in the enlicitide arm. Treatment discontinuation due to adverse events was rare (event rate: 0.007; 95% CI: 0.004–0.011; *p* < 0.0001; <1%), and all-cause mortality was low (event rate: 0.029; 95% CI: 0.023–0.036; *p* < 0.0001). No MACE data were available, as the CORALreef Outcomes trial (NCT06008756) remains ongoing.

### 3.6. Certainty of Evidence

**Figure 3:**
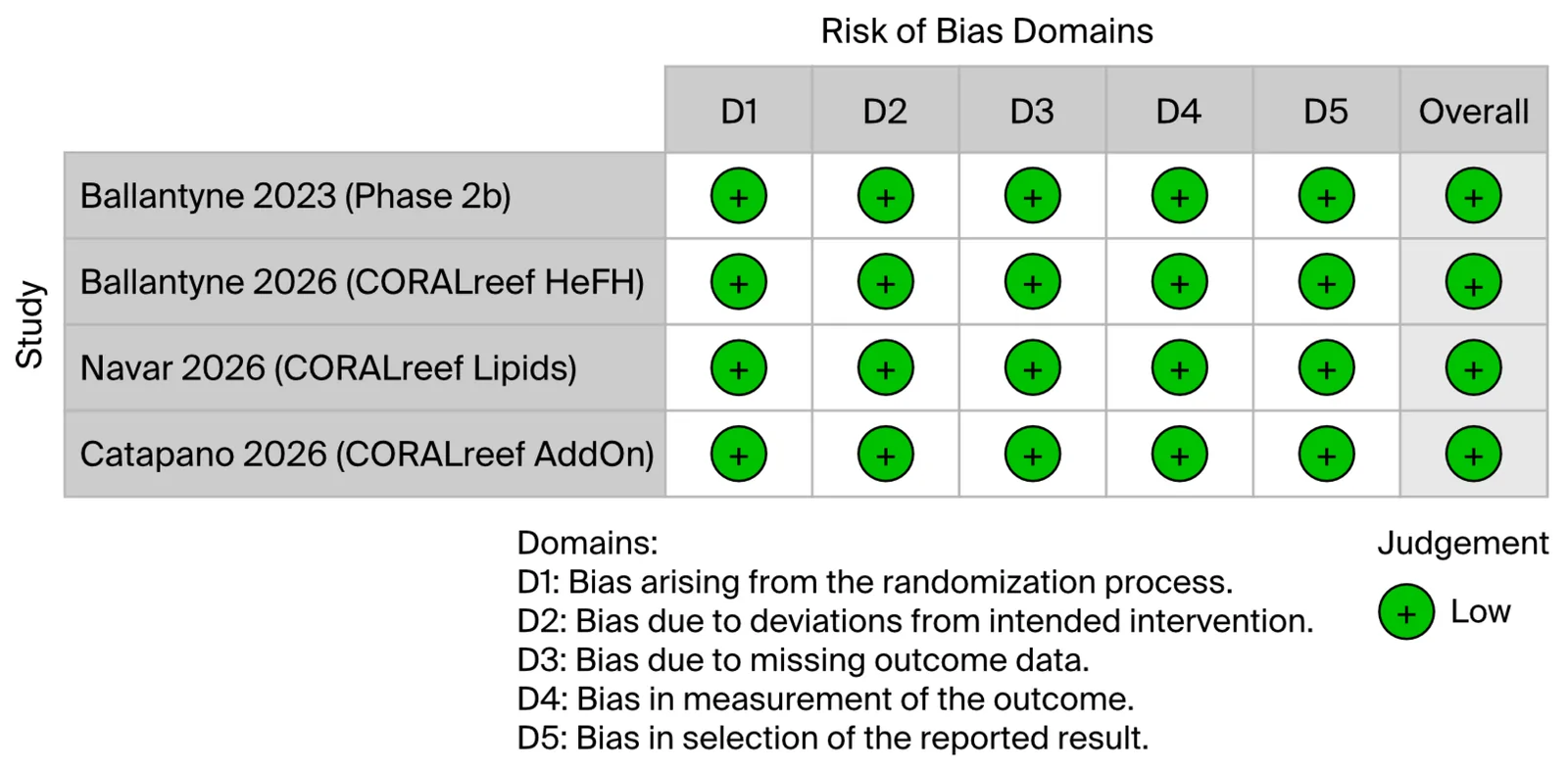
Cochrane RoB 2 risk-of-bias summary (traffic light plot) for the four included trials [8,9,13,20]. All studies were rated as low risk across domains D1–D5 and overall. D1, randomization process; D2, deviations from intended interventions; D3, missing outcome data; D4, measurement of the outcome; D5, selection of the reported result.

Under the GRADE framework, the certainty of evidence was rated low (⊕⊕◯◯) for all lipid efficacy outcomes, downgraded on two domains—inconsistency (I^2^ = 93.43–99.65%) and indirectness (surrogate endpoints without hard cardiovascular outcomes). Risk of bias was rated not serious, as none of the five Cochrane RoB 2 domains showed a concrete flaw; the single-sponsor nature of the evidence base was instead reflected once in publication bias to avoid double-counting (Figure 3). Safety outcomes were rated moderate (⊕⊕⊕◯), downgraded only for imprecision arising from low absolute event rates and relatively short follow-up. Evidence for major adverse cardiovascular events was rated very low, as no direct data were available. The GRADE Summary of Findings is detailed in Table 2.

### 3.7. Exploratory Meta-Regression on Dose

**Figure 4:**
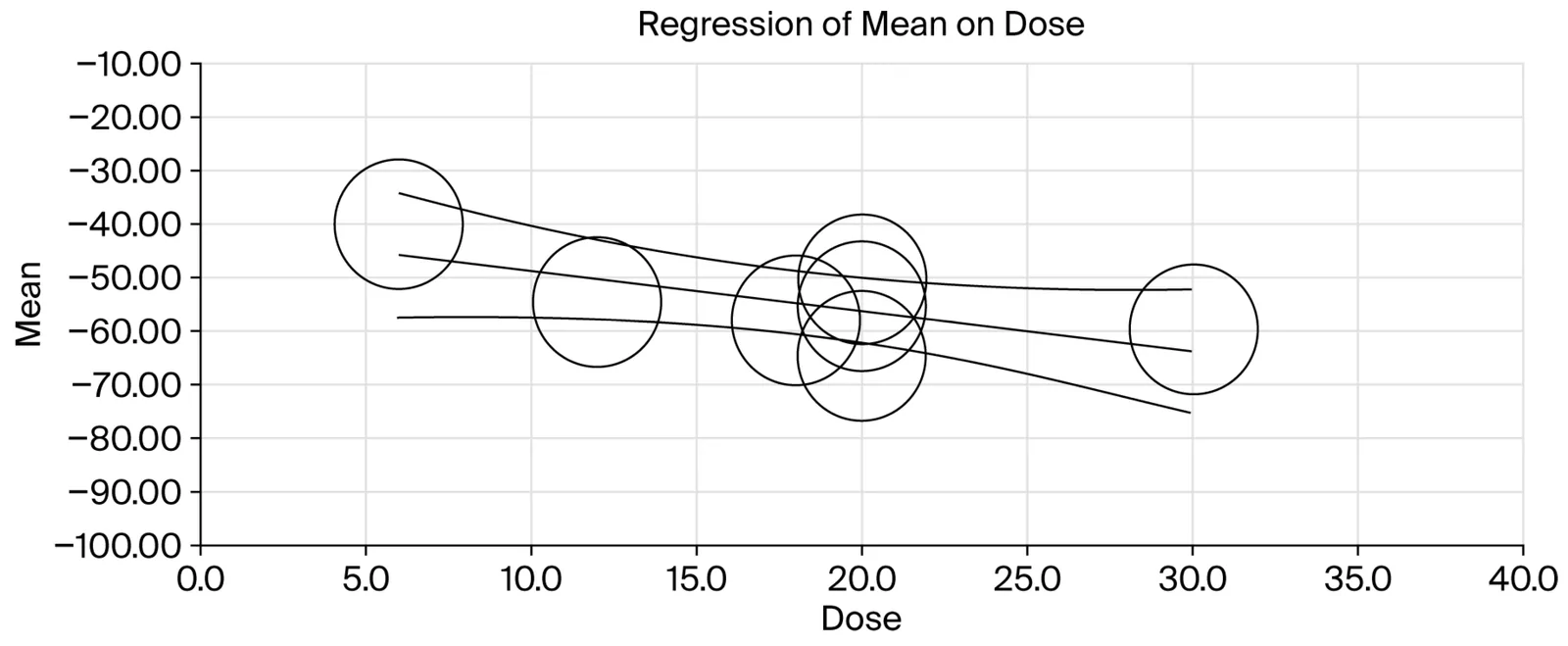
Random-effects meta-regression of percent change in LDL-C on enlicitide dose. Each circle represents an individual study/dose level, sized in proportion to its weight in the analysis; the solid line depicts the fitted regression slope with its 95% confidence band. A significant negative dose–response relationship was observed (β = −0.744 percentage points per mg; *p* = 0.028; R^2^ analog = 0.08).

A random-effects meta-regression of percent LDL-C change on enlicitide dose demonstrated a statistically significant negative association (slope β = −0.744 percentage points per mg; *p* = 0.028), confirming a dose-dependent effect (Figure 4). The model Q statistic was 4.80, while the residual Q (Q_E) remained large at 875.44, and the dose covariate accounted for only a modest proportion of between-study variance (R^2^ analog = 0.08). Correspondingly, the residual heterogeneity variance was only marginally reduced by inclusion of dose (τ^2^ decreasing from 41.47 in the null model to 37.98). Taken together, these findings indicate that while a significant dose–response relationship is present, dose alone explains only a small fraction (approximately 8%) of the substantial between-study heterogeneity; the remainder is likely attributable to differences in study-specific background lipid-lowering therapies, patient baseline characteristics, and follow-up durations. Furthermore, because the 6, 12, 18, and 30 mg doses were evaluated in the Phase 2b trial while the 20 mg dose was used in the Phase 3 trials, dose and study phase are substantially confounded. Thus, this meta-regression should be interpreted strictly as hypothesis-generating, with the within-trial arms of the Phase 2b study providing more direct, unconfounded evidence of dose-dependency. The four included trials enrolled a total of 3894 randomized participants and comprised one Phase 2b dose-ranging study [8] and three Phase 3 trials from the CORALreef program: CORALreef Lipids [13], CORALreef HeFH [9], and CORALreef AddOn [20]. Three trials used a placebo comparator, while CORALreef AddOn employed an active-comparator design against bempedoic acid, ezetimibe, and their combination. Baseline LDL-C ranged from 92.5 mg/dL in CORALreef AddOn to 119 mg/dL in the HeFH population. Background statin use was near-universal in the Phase 3 trials (96.6–100%). Enlicitide was administered orally once daily at 20 mg in all Phase 3 trials, and at 6–30 mg in the Phase 2b dose-ranging study. All trials were sponsored by Merck & Co., Inc. (Rahway, NJ, USA). The characteristics of the included trials are summarized in Table 1.

## 4. Discussion

### 4.1. Summary of Main Findings

This systematic review and meta-analysis provide the first quantitative synthesis of RCT evidence on enlicitide, an oral macrocyclic peptide PCSK9 inhibitor, in adults with hypercholesterolemia or HeFH, incorporating 52-week data from the two pivotal Phase 3 CORALreef trials. Across four trials enrolling 3894 participants, enlicitide achieved a clinically meaningful decrease in LDL-C (MD: −55.78 percentage points), along with significant reductions in ApoB, non-HDL-C, and Lp(a), and a consistently favorable safety profile. The durability of LDL-C lowering demonstrated at 52 weeks in the two largest trials is a particularly important finding, indicating that the effect is sustained with continuous oral administration.

### 4.2. Efficacy in Context: Benchmarking Against Existing Therapies

The magnitude of LDL-C reduction achieved by enlicitide (approximately 56%) substantially exceeds that of currently available oral non-statin therapies—ezetimibe (approximately 18–22% as monotherapy) and bempedoic acid (approximately 17–28%)—and is broadly comparable to that of injectable PCSK9 inhibitors (approximately 55–60% on top of statin therapy). This is a landmark observation: enlicitide appears to achieve injectable PCSK9 inhibitor-level efficacy through an oral route of administration, a goal that has eluded the field for over a decade since the discovery of the PCSK9 pathway. The head-to-head superiority of enlicitide over bempedoic acid, ezetimibe, and their combination in the active-comparator CORALreef AddOn trial further reinforces its position among oral agents [20,21].

Crucially, the therapeutic impact of enlicitide extends well beyond LDL-C reduction. As summarized in Table 2, concordant and clinically relevant decreases were achieved across the full spectrum of atherogenic lipid parameters. The observed −47.95 percentage point reduction in ApoB carries key clinical significance. Rather than relying solely on LDL-C, measuring ApoB provides a direct count of all circulating atherogenic particles, making it a more reliable predictor of cardiovascular risk in discordant phenotypes such as metabolic syndrome, diabetes, or hypertriglyceridemia [22]. The parallel reduction in non-HDL-C (−49.42 percentage points)—which captures cholesterol across all ApoB-containing lipoproteins—mirrors this finding and indicates that enlicitide reduces the overall atherogenic burden rather than lowering LDL-C in isolation. Notably, the close alignment of these three estimates (LDL-C, ApoB, and non-HDL-C), all clustering within a narrow 48–56% range, provides strong internal consistency. This symmetrical response strongly supports a coherent, receptor-mediated mechanism consistent with enhanced hepatic LDL receptor recycling following PCSK9 blockade [23].

The significant reduction in Lp(a) (−22.70 percentage points) merits close attention and represents a highly distinctive secondary finding. Lp(a) is an independent, largely genetically determined cardiovascular risk factor that remains essentially unresponsive to statin therapy and, in some analyses, is modestly increased by it [24]. Although monoclonal PCSK9 inhibitors reduce Lp(a) by approximately 20–30%, no oral lipid-lowering agent in current clinical use achieves a comparable effect. Consequently, the magnitude of Lp(a) reduction observed with enlicitide is of clinical interest, particularly for patients with elevated Lp(a) who currently lack approved oral therapeutic options. Dedicated RNA-targeted therapies like pelacarsen and olpasiran are highly potent but remain investigational and require subcutaneous injection [25,26]. Whether this reduction translates into a measurable reduction in cardiovascular events—independent of the concomitant LDL-C lowering—cannot be determined from the present data and warrants pre-specified evaluation in the ongoing CORALreef Outcomes trial.

Notably, CORALreef HeFH enrolled a substantial proportion of patients carrying LDLR variants [9]. Because enlicitide requires functional LDL receptors to exert its effects, patients with more severe loss-of-function LDLR genotypes may experience a smaller LDL-C reduction than those with residual receptor activity. This genotype-dependent variability may contribute to the observed heterogeneity and should be considered when generalizing the pooled estimate to severe familial phenotypes.

Taken together, the concordance across LDL-C, ApoB, and non-HDL-C, combined with a meaningful reduction in Lp(a), indicates that enlicitide delivers a comprehensive improvement in the atherogenic lipid profile. Nonetheless, all of these remain surrogate endpoints, and the certainty of this evidence was rated low under GRADE, principally on account of inconsistency and indirectness (Table 2). The translation of these favorable lipid effects into hard cardiovascular outcomes therefore remains to be established.

### 4.3. Interpretation of Heterogeneity

The considerable statistical heterogeneity observed across lipid efficacy outcomes (I^2^ range, 93.43–99.65%) requires careful contextual interpretation and must not be conflated with inconsistency in clinical effect—the direction of benefit was uniform across all trials. Several defensible sources of between-study variability are identifiable. First, the included trials span Phase 2b through Phase 3 development and incorporate a wide dose range (6–30 mg), with correspondingly different pharmacodynamic effects. Second, background lipid-lowering therapy differed markedly, from 60.6% statin use in the Phase 2b study to universal use in the Phase 3 trials. Third, the populations differed substantially, most notably between the HeFH cohort (baseline LDL-C 119 mg/dL) and the non-familial populations. Fourth, follow-up durations and primary-endpoint time points varied across trials.

Critically, leave-one-out sensitivity analyses confirmed that the direction and approximate magnitude of the primary effect remained stable after sequential omission of each study (I^2^ range, 65–90%). This consistency confirms that the overall efficacy estimate represents a collective trend rather than an artifact of a single dominant study. Our exploratory meta-regression provides direct empirical support for dose as one contributor to this heterogeneity: escalating doses of enlicitide were significantly associated with incremental lipid reductions (β = −0.744 per mg; *p* = 0.028). However, dose accounted for only approximately 8% of between-study variance, confirming that the bulk of heterogeneity arises from factors other than dose. In this context, the pre-specified use of a random-effects (DerSimonian–Laird) model was both appropriate and conservative, incorporating between-study variance rather than assuming a single common true effect. Given the limited range of dose cohorts evaluated, this exploratory relationship remains provisional and serves primarily to guide future clinical investigations rather than establish definitive dosing thresholds.

### 4.4. Safety Profile and Clinical Tolerability

The safety findings represent one of the most important contributions of this synthesis. The pooled event rate for serious adverse events (4.2%) is consistent with background rates expected in a high-cardiovascular-risk population, and the pooled incidence of any adverse event was low and did not reach statistical significance (*p* = 0.087). Treatment discontinuation due to adverse events was exceptionally rare (<1%), comparing very favorably with statins, for which real-world discontinuation rates of 5–10% are commonly reported [27]. These findings are further strengthened by the inclusion of 52-week data from the two largest Phase 3 trials, extending the safety observation window well beyond the 8–24 weeks typical of early-phase lipid trials. Concerns regarding gastrointestinal tolerability—a frequently cited barrier to the development of oral peptide therapeutics—are not substantiated by the pooled discontinuation data, suggesting acceptable tolerability at therapeutic doses.

In contrast to statins, ezetimibe, and bempedoic acid, PCSK9 inhibitors have generally not been shown to lower high-sensitivity C-reactive protein (hsCRP), an established marker of residual inflammatory risk [28]. None of the included enlicitide trials reported hsCRP, precluding synthesis of this outcome. Whether oral PCSK9 inhibition modifies inflammatory risk markers is an important question that future trials, including hsCRP as a pre-specified endpoint, should address.

### 4.5. Implications for Clinical Practice and Guidelines

Current ACC/AHA and ESC/EAS guidelines recommend PCSK9 inhibitor therapy for patients who do not achieve LDL-C targets on maximally tolerated statin therapy with or without ezetimibe. Yet uptake of injectable PCSK9 inhibitors remains suboptimal owing to needle aversion, access barriers, and reimbursement constraints. As an oral agent that achieves comparable LDL-C lowering with durable 52-week efficacy, enlicitide directly addresses each of these barriers. Three underserved populations stand to benefit in particular: patients with statin intolerance requiring greater LDL-C reduction than ezetimibe or bempedoic acid can provide; patients with HeFH in whom injectable PCSK9 inhibitor therapy is indicated but declined; and patients in resource-limited settings where cold-chain storage and injection infrastructure are impractical [2,29].

### 4.6. Strengths and Limitations

The principal strengths of this review include its comprehensive multi-database search, rigorous PRISMA 2020–concordant methodology, duplicate screening and data verification in Covidence, and the incorporation of the most recent 52-week Phase 3 efficacy and safety data. Several limitations must nonetheless be acknowledged. First, all included trials were funded by a single sponsor (Merck & Co., Inc., Rahway, NJ, USA), raising the possibility of publication and reporting bias, which cannot be excluded without independently funded replication studies. Second, the small number of included trials (*n* = 4) precluded formal funnel-plot–based assessment of publication bias and limited the feasibility of some pre-specified subgroup analyses. Third, considerable heterogeneity across lipid outcomes reduces the precision of pooled estimates, although sensitivity analyses supported their robustness. Fourth, all efficacy outcomes were surrogate endpoints; the absence of hard cardiovascular outcome data means that certainty of evidence for efficacy was low by GRADE, and clinical-event benefit cannot yet be inferred. Fifth, although the two largest trials contributed 52-week data, the remaining studies had shorter follow-up, limiting conclusions regarding long-term safety. Sixth, the exploratory dose meta-regression was limited by near-complete confounding between dose and study phase: all non–20 mg dose levels (6, 12, 18, and 30 mg) were contributed by the randomized dose arms of a single Phase 2b trial, whereas the 20 mg dose derived exclusively from the Phase 3 trials in distinct populations. Consequently, the observed dose–response slope cannot be fully disentangled from differences in population, background therapy, and study design across phases, and the within-trial dose arms of the Phase 2b study should be regarded as the more direct basis for inferring dose-dependency.

### 4.7. Future Research Directions

The results of the CORALreef Outcomes trial (NCT06008756; *N* ≈ 14,500; estimated completion ~2028) will be pivotal in determining whether the robust and durable LDL-C lowering observed with enlicitide translates into a reduction in hard cardiovascular events; future meta-analyses should incorporate these data when available. Independent, non-industry-funded replication studies across diverse geographic and ethnic populations are needed to confirm generalizability; head-to-head trials against injectable PCSK9 inhibitors would provide the comparative effectiveness data required to define enlicitide’s optimal clinical positioning.

Beyond LDL-C lowering, several determinants of cardiovascular risk reduction remain uncharacterized for enlicitide. The available trials did not report effects on inflammatory markers such as hsCRP, and the durability of particle-level and Lp(a) benefits, as well as their translation into event reduction, remains to be established. These represent priority endpoints for the ongoing CORALreef Outcomes trial and for future dedicated studies.

## 5. Conclusions

Enlicitide produces robust, durable, and consistent reductions in LDL-C, ApoB, non-HDL-C, and Lp(a) in adults with hypercholesterolemia and HeFH, with a favorable safety and tolerability profile sustained across a 52-week treatment period. The magnitude of LDL-C lowering achieved by this oral macrocyclic peptide PCSK9 inhibitor is comparable to that of injectable PCSK9 inhibitors, while offering the transformative advantage of oral administration. These findings position enlicitide as a potentially paradigm-shifting therapy for patients with hypercholesterolemia—particularly those with statin intolerance, needle aversion, or barriers to parenteral therapy. Cardiovascular outcome data from the ongoing CORALreef Outcomes trial are essential to establish definitive clinical benefit and to support integration of enlicitide into cardiovascular risk-reduction guidelines.

The findings and their implications should be discussed in the broadest context possible. Future research directions may also be highlighted.

## Figures and Tables

**Table 1:** Characteristics of the four randomized controlled trials of enlicitide included in the meta-analysis. (**A**) Study design and baseline population characteristics. (**B**) Efficacy and safety outcomes.

(**A**)								
**Trial (First Author, Year)**	**Phase and Design**	**Sites/Countries**	**Key Inclusion Criteria**		**Treatment Arms and Randomization Ratio**		**N**	**Baseline Characteristics**
Phase 2b Ballantyne et al., 2023 [8] NCT05261126	Phase 2b, multicenter, double-blind, placebo-controlled, dose-ranging RCT	63 sites, 8 countries	Adults 18–80 y with hypercholesterolemia across the ASCVD risk spectrum (clinical ASCVD, intermediate/high risk, or borderline risk); LDL-C above risk-specific thresholds; fasting TG < 400 mg/dL. HoFH excluded.		Enlicitide 6, 12, 18 or 30 mg once daily vs. matching placebo (1:1:1:1:1)		381 (380 treated)	Median age 62 y; 49.3% female; mean LDL-C 119.5 ± 34.8 mg/dL; statin: none 39.4%, low–moderate 34.6%, high 26.0%; ezetimibe 13.9%; clinical ASCVD 38.6%; HeFH 7.6%
CORALreef Lipids Navar et al., 2026 [13] NCT05952856	Phase 3, multinational, double-blind, placebo-controlled RCT	168 sites, 14 countries	Adults ≥ 18 y with prior major ASCVD event and LDL-C ≥ 55 mg/dL, or at intermediate-to-high risk of a first ASCVD event and LDL-C ≥ 70 mg/dL; on stable lipid-lowering therapy including ≥ moderate-intensity statin (unless statin-intolerant); fasting TG < 400 mg/dL.		Enlicitide 20 mg once daily vs. matching placebo (2:1)		2909 (ITT)	Mean age 62.8 ± 10.7 y; 39.3% female; 53.9% White, 26.3% Asian; mean LDL-C 96.1 ± 38.9 mg/dL; statin 96.6% (moderate/high 95.4%); ezetimibe or hybutimibe 25.8%; prior major ASCVD 58.3%
CORALreef HeFH Ballantyne et al., 2026 [9] NCT05952869	Phase 3, multinational, double-blind, placebo-controlled, parallel-group RCT	59 sites, 17 countries	Adults ≥ 18 y with HeFH (genetic diagnosis or validated clinical algorithm) on ≥moderate-intensity statin; LDL-C ≥ 55 mg/dL with prior major ASCVD or ≥70 mg/dL without; fasting TG ≤ 400 mg/dL. HoFH excluded.		Enlicitide 20 mg once daily vs. matching placebo (2:1)		303	Mean age 52.4 ± 13.5 y; 51% female; mean LDL-C 119.0 ± 41.0 mg/dL; statin 100% (high-intensity 81.5%); ezetimibe 64.4%; prior major ASCVD 26.7%; known HeFH variant 58.4% (LDLR 51.2%)
CORALreef AddOn Catapano et al., 2026 [20] NCT06450366	Phase 3, multicenter, double-blind, active comparator, parallel-group RCT	35 sites, 8 countries	Statin-treated adults ≥ 18 y with prior major ASCVD event and LDL-C ≥ 55 mg/dL, or at intermediate-to-high risk of a first event and LDL-C ≥ 70 mg/dL; fasting TG < 400 mg/dL. HoFH, compound/double HeFH and concurrent PCSK9i excluded.		Enlicitide 20 mg vs. bempedoic acid 180 mg vs. ezetimibe 10 mg vs. bempedoic acid + ezetimibe (2:1:1:2)		301	Mean age 64.4 ± 10.1 y; 37.2% female; 65.8% White, 26.6% Asian; mean LDL-C 91.6 ± 30.3 mg/dL; statin 100% (moderate/high 97.6%; high-intensity 46.8%); prior major ASCVD 44.2%; HeFH 5.0%
(**B**)								
**Trial**	**Treatment Duration/Primary Time Point**	**Primary Efficacy Outcome (% Change in LDL-C)**		**Key Secondary Lipid Outcomes (Within-Arm Change from Baseline)**		**LDL-C Goal Attainment**		**Safety**
Phase 2b (Ballantyne 2023) [8]	8-week treatment +8-week safety follow-up (16 weeks total); Week 8	Placebo-adjusted LDL-C reduction at Week 8: −41.2% (6 mg; 95% CI −47.8 to −34.7) −55.7% (12 mg; −62.3 to −49.1) −59.1% (18 mg; −65.7 to −52.5) −60.9% (30 mg; −67.6 to −54.3) All *p* < 0.001 vs. placebo		ApoB: −32.8% to −51.8% (6→30 mg) Non-HDL-C: −35.9% to −55.8% Lp(a) (exploratory): −12.2% to −23.7% Dose-dependent across all parameters		Protocol-defined LDL-C goal: 80.5/85.5/90.8/90.8% vs. 9.3% placebo		Any AE 44.2/39.5/43.4/42.1% vs. 44.0% (placebo) Serious AE 1.3/3.9/2.6/2.6% vs. 0% D/C due to AE ≤ 2 participants per arm (2.6/0/2.6/2.6% vs. 1.3%) 1 death (18 mg arm; road-traffic accident, unrelated)
CORALreef Lipids (Navar 2026) [13]	52 weeks Week 24 (primary) Week 52 (key secondary)	Week 24: −57.1% vs. +3.0% (placebo) Between-group difference −55.8 pp (95% CI −60.9 to −50.7); *p* < 0.001 Week 52: difference −47.6 pp (−52.7 to −42.5); *p* < 0.001 Post hoc reanalysis: −59.7 pp (Week 24)		Week 24 (placebo-adjusted): Non-HDL-C −53.4 pp (−55.5 to −51.2) ApoB −50.3 pp (−52.1 to −48.5) Lp(a) −28.2 pp (−30.3 to −26.0) All *p* < 0.001 Week 52 (exploratory): −50.9/−47.2/−28.5 pp		LDL-C < 70 mg/dL and ≥ 50% ↓: 70.3% vs. 1.5% LDL-C < 55 mg/dL and ≥ 50% ↓: 67.5% vs. 1.2%		Any AE 64.3% vs. 62.1% Serious AE 9.9% vs. 12.0% D/C due to AE 3.1% vs. 4.1% Death 0.7% vs. 0.7% New/worsening diabetes 6.1% vs. 5.8% Drug-induced liver injury: 0 in both arms
CORALreef HeFH (Ballantyne 2026) [9]	52 weeks; Week 24 (primary) Week 52 (key secondary)	Week 24: −58.2% vs. +2.6% (placebo) Between-group difference −59.4 pp (95% CI −65.6 to −53.2); *p* < 0.001 Week 52: −55.3% vs. +8.7%; difference −61.5 pp (−69.4 to −53.7); *p* < 0.001		Week 24 (placebo-adjusted): Non-HDL-C −53.0 pp (−58.5 to −47.4) ApoB −49.1 pp (−54.0 to −44.3) Lp(a) −27.5 pp (−34.3 to −20.6) All *p* < 0.001 Week 52: −58.1/−51.8/−29.6 pp		LDL-C < 70 mg/dL and ≥ 50% ↓: 70.8% vs. 1.0% LDL-C < 55 mg/dL and ≥ 50% ↓: 67.3% vs. 1.0%		Any AE 77.7% vs. 76.2% Serious AE 4.5% vs. 4.0% (no intervention-related) D/C due to AE 2.0% vs. 3.0% 1 death (enlicitide; ischemic stroke, adjudicated unrelated) New/worsening diabetes 2.0% vs. 3.0%
CORALreef AddOn (Catapano 2026) [20]	56 days; Day 56	Day 56 LDL-C: −64.6% (enlicitide) vs. −6.3% (bempedoic acid), −27.8% (ezetimibe), −36.5% (bempedoic acid + ezetimibe) Adjusted differences −56.7/−36.0/ −28.1 pp; all *p* < 0.001 (superiority)		ApoB −54.6% (enlicitide); differences −47.5/−33.6/−26.8 pp, *p* < 0.001 Non-HDL-C −58.0%; differences −51.2/−32.2/−25.8 pp, *p* < 0.001 Lp(a) −26.2% (not multiplicity-controlled); differences −40.3/−26.2/−41.4 pp vs. bempedoic acid, ezetimibe, and their combination, respectively.”		LDL-C < 70 mg/dL and ≥ 50% ↓: 81.2% vs. 2.0/ 8.0/22.0% LDL-C < 55 mg/dL and ≥ 50% ↓: 78.2% vs. 2.0/ 8.0/20.0%		Any AE 40% (enlicitide) vs. 38/36/45% Serious AE 0% with enlicitide vs. 8/2/0% D/C due to AE 2% vs. 4/0/4% No deaths; no drug-induced liver injury; no new-onset or worsening diabetes

ASCVD, atherosclerotic cardiovascular disease; HeFH, heterozygous familial hypercholesterolemia; HoFH, homozygous familial hypercholesterolemia; ITT, intention-to-treat; LDL-C, low-density lipoprotein cholesterol; PCSK9i, proprotein convertase subtilisin/kexin type 9 inhibitor; RCT, randomized controlled trial; TG, triglycerides. All four trials were sponsored by Merck Sharp & Dohme LLC, a subsidiary of Merck & Co., Inc. (Rahway, NJ, USA). Enlicitide was administered orally once daily in the fasted state in every trial. AE, adverse event; ApoB, apolipoprotein B; CI, confidence interval; D/C, discontinuation; Lp(a), lipoprotein(a); non-HDL-C, non-high-density lipoprotein cholesterol; pp, percentage points. ↓, indicates reduction.

**Table 2:** GRADE Summary of Findings—enlicitide versus placebo or active comparator.

Outcome	Risk of Bias	Inconsistency	Indirectness	Imprecision	Pub. Bias	Pooled Effect (95% CI)	Certainty
LDL-C % change (Primary)	Not serious ^1^	Serious ^2^	Serious ^3^	Not serious	Flagged ^5^	MD −55.78 pp (−61.15 to −50.42); *p* < 0.0001; I^2^ = 99.58%	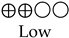
ApoB % change	Not serious ^1^	Serious ^2^	Serious ^3^	Not serious	Flagged ^5^	MD −47.95 pp (−51.22 to −44.67); *p* < 0.0001; I^2^ = 99.25%	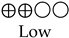
Non-HDL-C % change	Not serious ^1^	Serious ^2^	Serious ^3^	Not serious	Flagged ^5^	MD −49.42 pp (−54.23 to −44.60); *p* < 0.0001; I^2^ = 99.65%	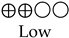
Lp(a) % change	Not serious ^1^	Serious ^2^	Serious ^3^	Not serious	Flagged ^5^	MD −22.70 pp (−28.62 to −16.78); *p* < 0.0001; I^2^ = 93.43%	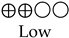
Serious adverse events	Not serious ^1^	Not serious	Not serious	Serious ^4^	Flagged ^5^	ER 0.042 (0.017–0.101); *p* < 0.0001	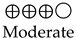
Any adverse events	Not serious ^1^	Not serious	Not serious	Serious ^4^	Flagged ^5^	ER 0.21 (0.05–0.54); *p* = 0.087	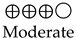
Discontinuation due to AE	Not serious ^1^	Not serious	Not serious	Serious ^4^	Flagged ^5^	ER 0.007 (0.004–0.011); *p* < 0.0001	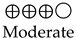
All-cause mortality	Not serious ^1^	Not serious	Not serious	Serious ^4^	Flagged ^5^	ER 0.029 (0.023–0.036); *p* < 0.0001	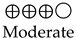
MACE	—	—	—	—	—	No data (CORALreef Outcomes ongoing)	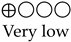

Footnotes: ^1^ Although all trials were industry-funded (Merck & Co., Inc., Rahway, NJ, USA), none of the five Cochrane RoB 2 domains showed concrete flaws; single-sponsor funding is not itself a RoB 2 domain and, to avoid double-counting, is reflected once under publication bias rather than under risk of bias. ^2^ Considerable statistical inconsistency (I^2^ = 93.43–99.65%); downgraded by one level, as the direction of effect was consistent across trials. ^3^ LDL-C and related lipids are surrogate endpoints; no cardiovascular outcome data available. ^4^ Low absolute event rate with relatively short follow-up. ^5^ Publication bias could not be formally assessed (*n* = 4) and is flagged narratively given the single-sponsor evidence base with uniformly positive results, not separately scored. ApoB, apolipoprotein B; CI, confidence interval; ER, event rate; LDL-C, low-density lipoprotein cholesterol; Lp(a), lipoprotein(a); MACE, major adverse cardiovascular event; MD, mean difference; pp, percentage points.

## Data Availability

All data analyzed in this review are derived from published sources and are available within the article. No new primary data were created or analyzed in this study. All data analyzed during this systematic review and meta-analysis are derived from publicly available, previously published randomized controlled trials cited within the manuscript.

## Supplementary Materials

The following supporting information can be downloaded at https://www.mdpi.com/article/10.3390/jcdd13080398/s1: PRISMA 2020 Main Checklist [10].

## Abbreviations

The following abbreviations are used in this manuscript:

AE	Adverse event
ApoB	Apolipoprotein B
ASCVD	Atherosclerotic cardiovascular disease
HDL-C	High-density lipoprotein cholesterol
HeFH	Heterozygous familial hypercholesterolemia
LDL-C	Low-density lipoprotein cholesterol
Lp(a)	Lipoprotein(a)
PCSK9	Proprotein convertase subtilisin/kexin type 9
